# *Pathogens* Best Paper Awards for 2015

**DOI:** 10.3390/pathogens4020387

**Published:** 2015-06-15

**Authors:** Lawrence S. Young

**Affiliations:** Pro-Vice Chancellor, Warwick Medical School, University of Warwick, Coventry CV4 7AL, UK; E-Mail: L.S.Young@warwick.ac.uk

*Pathogens* is instituting annual awards to recognize the most outstanding papers in the areas of all aspects of pathogens and pathogen-host interactions published in *Pathogens*.

We are pleased to announce the recipients of the first “*Pathogens* Best Paper Awards”. Nominations were selected by the Editor-in-Chief and several Editorial Board members of *Pathogens* from all original research articles published in 2013 or 2014.

We are pleased to announce that the following three papers were chosen to receive “*Pathogens* Best Paper Awards” for 2015. I congratulate all of the authors and thank them for their significant and important contributions to the field of pathogens. I also thank the members of the Selection Committee for their efforts.

**Article Award**

First Prize
  **Eric W. Rogier, Aubrey L. Frantz, Maria E. C. Bruno and Charlotte S. Kaetzel**
  Secretory IgA is Concentrated in the Outer Layer of Colonic Mucus along with Gut Bacteria
  *Pathogens* **2014**, *3*(2), 390-403; doi:10.3390/pathogens3020390
  Available online: http://www.mdpi.com/2076-0817/3/2/390
Second Prize
  **Anthony F. Barbet, Basima Al-Khedery, Snorre Stuen, Erik G. Granquist, Roderick F. Felsheim and Ulrike G. Munderloh**
  An Emerging Tick-Borne Disease of Humans Is Caused by a Subset of Strains with Conserved Genome Structure
  *Pathogens* **2013**, *2*(3), 544-555; doi:10.3390/pathogens2030544
  Available online: http://www.mdpi.com/2076-0817/2/3/544
Third Prize
  **Philippe Gérard**
  Metabolism of Cholesterol and Bile Acids by the Gut Microbiota
  *Pathogens* **2014**, *3*(1), 14-24; doi:10.3390/pathogens3010014
  Available online: http://www.mdpi.com/2076-0817/3/1/14

***Award Selection Committee***

Editor-in-Chief
  **Prof. Dr. Lawrence S. Young**
  Pro-Vice Chancellor, Warwick Medical School, University of Warwick, Coventry CV4 7AL, UK
  E-Mail: L.S.Young@warwick.ac.uk
Editorial Board Member
  **Dr. Jean-Pierre Gorvel**
  Centre d'immunologie de Marseille Luminy, Case 906, 13288 Marseille, CEDEX 09, France
  E-Mail: gorvel@ciml.univ-mrs.fr
Editorial Board Member
  **Prof. Dr. Moriya Tsuji**
  HIV and Malaria Vaccine Program, Aaron Diamond AIDS Research Center, Affiliate of The Rockefeller University, 455 First Ave, Room 747, New York, NY 10016, USA
  E-Mail: mtsuji@adarc.org
Editorial Board Member
  **Prof. Dr. Howard F. Jenkinson**
  University of Bristol, School of Oral and Dental Sciences, Lower Maudlin Street, Bristol, BS1 2LY, UK
  E-Mail: Howard.Jenkinson@bristol.ac.uk
Editorial Board Member
  **Prof. Dr. Jean E. Crabtree**
  Molecular Gastroenterology Section, Leeds Institute of Molecular Medicine, Wellcome Trust Brenner Building, St. James's University Hospital, Leeds LS9 7TF, UK
  E-Mail: J.Crabtree@leeds.ac.uk
Editorial Board Member
  **Dr. Frank Lafont**
  Center of Infection and Immunity of Lille, Institut Pasteur de Lille-CNRS UMR8204-INSERM U1019- 1 rue du Prof Calmette, F-59021 Lille, France
  E-Mail: frank.lafont@pasteur-lille.fr

